# Differentiating Avian and Seasonal Influenza Viruses in Wastewater by Digital PCR

**DOI:** 10.3201/eid3211.261234

**Published:** 2026-11

**Authors:** Valentina Paracchini, Mauro Petrillo, Francisco Mayo Leon, Gerhard Buttinger, Sabrina Marciano, Marika Crimaudo, Calogero Terregino, Carolina Veneri, Giuseppina La Rosa, Elisabetta Suffredini, Clementina Cocuzza, Bernd M. Gawlik, Tobias Wiesenthal, Ursula Vincent, Valentina Panzarin, Antonio Marchini

**Affiliations:** European Commission, Joint Research Centre, Geel, Belgium (V. Paracchini, F.M. Leon, G. Buttinger, U. Vincent); Seidor Italy Srl., Milan, Italy (M. Petrillo); Istituto Zooprofilattico Sperimentale delle Venezie, Legnaro, Italy (S. Marciano, M. Crimaudo, C. Terregino, V. Panzarin); Istituto Superiore di Sanità, Rome, Italy (C. Veneri, G. La Rosa, E. Suffredini); University of Milano-Bicocca, Monza, Italy (C. Cocuzza); European Commission, Joint Research Centre, Ispra, Italy (B.M. Gawlik, T. Wiesenthal, A. Marchini)

**Keywords:** Avian influenza, influenza, HPAI A(H5N1) clade 2.3.4.4b, digital RT-PCR, wastewater surveillance, seasonal influenza, viruses, public health, influenza A virus, influenza virus monitoring, respiratory infections, Italy

## Abstract

Highly pathogenic avian influenza A(H5Nx) viruses, particularly clade 2.3.4.4b, pose substantial global health risks. We combined and piloted 2 duplex reverse transcription digital PCRs to discriminate between avian and seasonal influenza viruses in wastewater. This approach enabled rapid, strain-specific detection to reduce ambiguity in environmental surveillance for influenza viruses.

Highly pathogenic avian influenza (HPAI) A(H5Nx) viruses, particularly clade 2.3.4.4b, continue to circulate widely among wild and domestic birds, with recurrent spillover into mammalian species and sporadic infections in humans. The extensive geographic distribution and persistent circulation of clade 2.3.4.4b, together with its predominance among HPAI A(H5) viruses currently circulating in Europe, support its continued relevance for animal and public health surveillance (*1*–*4*).

Wastewater and environmental surveillance (WES) provides a population-level complement to clinical, veterinary, and wildlife surveillance and is increasingly integrated into the monitoring framework in Europe (*5*). WES has been widely used for SARS-CoV-2 and has also been applied to influenza A and B viruses (*6*,*7*).

WES typically relies on PCR-based methods to detect and quantify pathogens. Similar to other pathogens, influenza RNA concentrations in wastewater might vary according to shedding patterns, sewer inputs, matrix characteristics, and the relative contributions of human and animal sources (*6*). Differences in laboratory methods and reporting further complicates interpretation, underscoring the necessity of considering assay sensitivity and specificity when comparing results across studies (*8*).

The matrix gene is the preferred molecular target for the screening of influenza A viruses (*9*) because it is conserved across seasonal human and animal influenza strains, but broad reactivity limits strain discrimination in mixed environmental samples. For instance, the influenza A component of the Centers for Disease Control and Prevention (CDC) influenza SARS-CoV-2 (Flu SC2) multiplex reverse transcription PCR (RT-PCR), developed for clinical respiratory specimens (*10*) and adapted for digital RT-PCR (dRT-PCR) analysis of wastewater samples (*11*), can detect both seasonal human and avian influenza A signals (*12*).

Several wastewater assays were designed to detect H5 viruses (*13**–**15*). Those assays are useful for broad H5 virus screening but might require further characterization to distinguish between HPAI H5N1 clade 2.3.4.4b, another H5 lineage, or low pathogenicity avian influenza. A clade-specific assay can provide additional epidemiologic resolution and help prioritize follow-up across animal and human surveillance.

We previously developed the European Commission’s Joint Research Centre (JRC) RT-PCRs targeting the hemagglutinin (HA) and matrix protein (MP) genes. The JRC-HA assay targets a conserved HA region in HPAI H5N1 clade 2.3.4.4b and the JRC-MP assay targets the conserved matrix gene for broader detection of influenza A (*12*) (Appendix). We evaluated a dual-duplex dRT-PCR approach combining the CDC influenza A and B assay components with the JRC-HA and JRC-MP assay to discriminate between avian and seasonal human influenza viruses in wastewater.

## The Study

We first performed a comparative in silico analysis of the publicly available primer and probe sequences from 2 H5 assays (*13*–*15*), and the JRC-HA and JRC-MP assays (*12*) (Figure 1). The predicted detection of HPAI H5 clade 2.3.4.4b was broadly comparable across the assays, but the JRC-HA assay revealed greater discrimination against non-2.3.4.4b HPAI clades (Figure 1, panel A). The JRC-HA and JRC-MP assays had the lowest predicted cross-reactivity with low pathogenicity avian influenza H5 annotated sequences (Figure 1, panels A, B).

**Figure 1:**
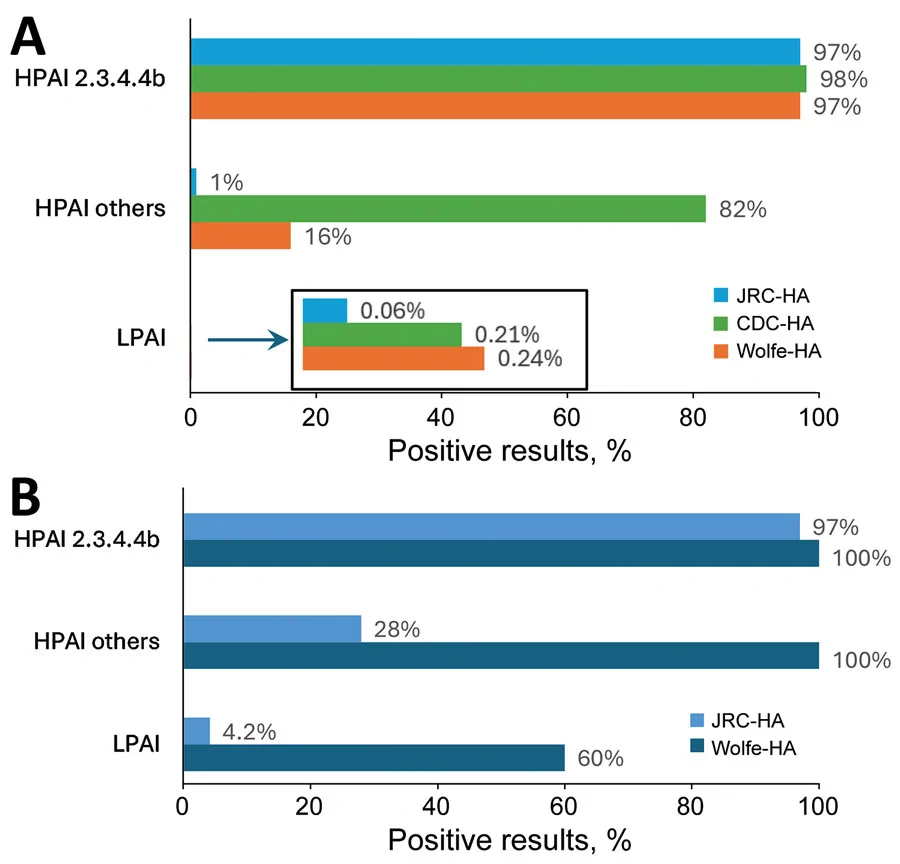
In silico comparative analysis of wastewater assays used to detect avian and seasonal influenza viruses in wastewater. A) Predicted detection of HA gene sequences from LPAI viruses, HPAI viruses other than clade 2.3.4.4b, and HPAI clade 2.3.4.4b viruses by the Wolfe-HA (*13*), CDC-HA *(**14*,*15**)*, and JRC-HA (*12*) assays. Bars indicate the percentage of sequences predicted to yield positive results. Inset box represents enlarged results for LPAI. B) Predicted detection of MP gene sequences from LPAI viruses, HPAI viruses other than clade 2.3.4.4b, and HPAI clade 2.3.4.4b viruses by the Wolfe-MP (*13*), and JRC-MP (*12*) assays. Bars indicate the percentage of sequences predicted to yield positive results. CDC, Centers for Disease Control and Prevention; HPAI, highly pathogenic avian influenza; HA, hemagglutinin; JRC, Joint Research Centre; LPAI, low pathogenicity avian influenza; MP, matrix protein.

Then, we evaluated the JRC-HA and JRC-MP duplex assay by using HPAI H5N1 clade 2.3.4.4b viruses collected from wild birds and poultry in northern Italy during October–December 2025 (Appendix Table 4). Sequence alignment revealed high conservation across the primer and probe binding regions for both assays, indicating that recent viral evolution had not substantially affected assay inclusivity. Nevertheless, we identified 2 substitutions within the JRC-HA probe-binding region in 3 samples (Appendix Figure 1). In silico PCR predicted amplification (data not shown), and both quantitative RT-PCR and dRT-PCR detected influenza A in all 10 samples, including samples with low viral concentrations (Figure 2, panels A, B). For samples H, I, and J, lowering the JRC-HA/JRC-MP dRT-PCR annealing temperature from 60°C to 56.4°C improved partition separation (Appendix Figure 2). At the revised annealing temperature, in silico analysis indicated that stable primer-dimer formation was unlikely, and assay specificity was not affected (Figure 2, panels C–F). The limit of detection was 5 copies/reaction, consistent with previous results (*12*). The CDC influenza B assay failed to detect the target in any of the 10 samples analyzed, whereas the CDC influenza A assay detected the target in all 10 samples with comparable performance to that of the JRC-HA and JRC-MP assays (Figure 2, panels A, B). Such broad detection is useful for confirming the presence of influenza virus; however, it does not permit discrimination between avian and seasonal human influenza A viruses in mixed specimens such as wastewater samples collected during periods of seasonal influenza circulation.

**Figure 2:**
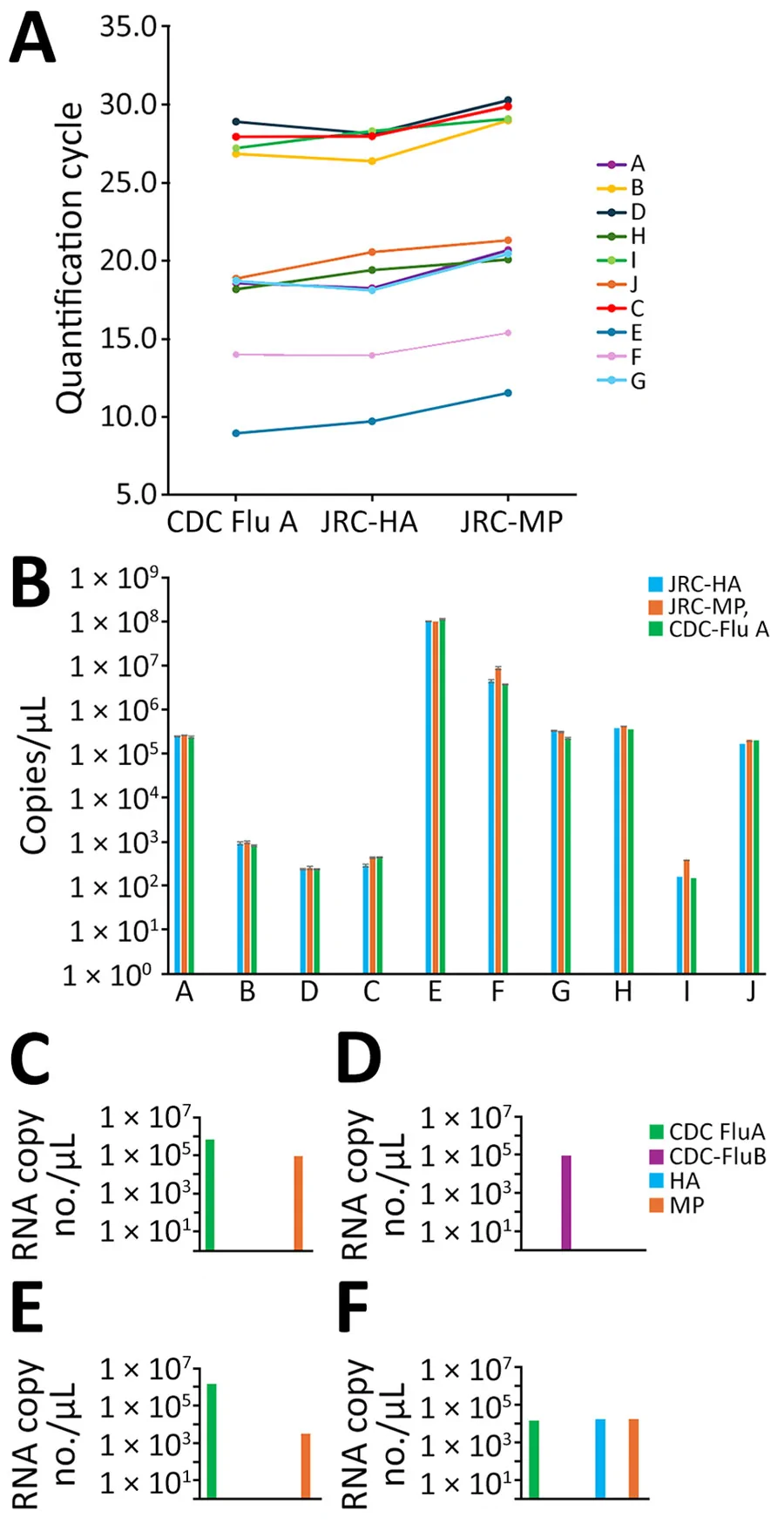
Analytical evaluation of JRC-HA and JRC-MP RT-PCRs against recent highly pathogenic avian influenza (HPAI) H5N1 clade 2.3.4.4b isolates for differentiating avian and human influenza A viruses from wastewater samples. A) Quantification cycle values for 10 HPAI H5N1 clade 2.3.4.4b clinical samples from Italy measured by using the JRC-HA, JRC-MP, and CDC influenza A quantitative reverse transcription PCRs. B) Viral RNA copy number estimates for 10 HPAI H5N1 clade 2.3.4.4b clinical samples from Italy measured by using the JRC-HA, JRC-MP, and CDC influenza A reverse transcription digital PCR assays. C–F) Verification of assay specificity of the JRC-HA and JRC-MP duplex assay at the annealing temperature of 56.4°C. Assay specificity was evaluated in digital PCR, compared with the influenza A and B components of the CDC influenza SARS-CoV-2 multiplex assay, by measuring the RNA copy numbers per microliter for influenza A(H1N1) (C), influenza B (D), influenza A(H3N2) synthetic RNAs (from Twist Bioscience, https://www.twistbioscience.com) (E), and HPAI H5N1 clade 2.3.4.4b RNA isolated from an infected animal (F). CDC, Centers for Disease Control and Prevention; HA, hemagglutinin; JRC, Joint Research Centre; MP, matrix protein.

To improve discrimination between avian and seasonal human influenza in wastewater samples, we evaluated the influenza A and B components of the CDC Flu SC2 multiplex assay together with the JRC-HA and JRC-MP duplex assay. We performed the 2 duplex assays separately, according to the conditions described previously (*12*) (Appendix). Because HPAI H5N1 clade 2.3.4.4b–positive wastewater samples were unavailable, we added defined amounts of avian and seasonal human influenza A RNA to RNA extracts from influenza-negative wastewater samples (Figure 3). We evaluated the 2 duplex assays under 3 simulated co-circulation scenarios.

**Figure 3:**
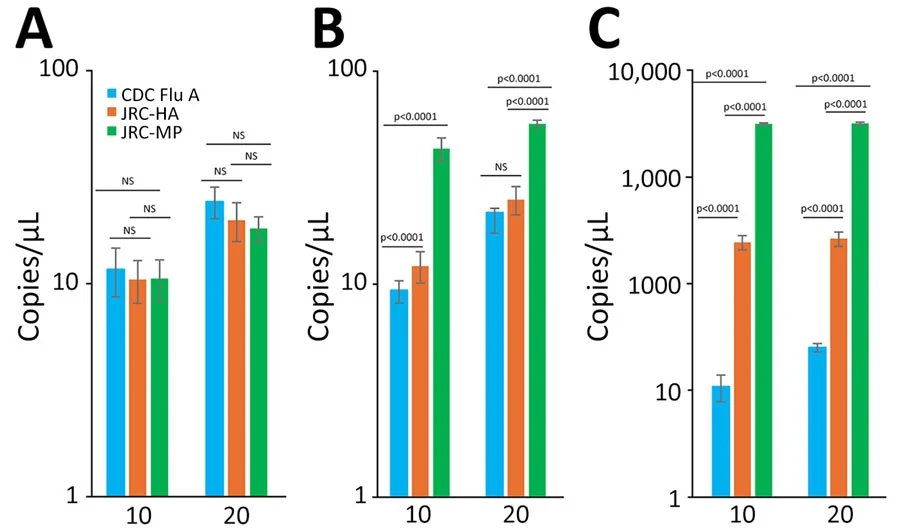
Detection of influenza A and highly pathogenic avian influenza (HPAI) A(H5N1) in simulated wastewater samples by using reverse transcription digital PCR. A) Copy numbers of influenza A virus in wastewater samples negative for both avian and human influenza A spiked with 10 or 20 copies/µL of HPAI H5N1 clade 2.3.4.4b virus. B) Copy number of influenza A virus in wastewater samples negative for both avian and human influenza A spiked with 10 or 20 copies/µL of HPAI H5N1 clade 2.3.4.4b virus and 35 copies/µL of human influenza A virus. C) Copy number of influenza A virus in wastewater samples negative for both avian and human influenza A spiked with 10 or 20 copies/µL of HPAI H5N1 clade 2.3.4.4b virus and 3,500 copies/µL of human influenza A virus. Error bars indicate the mean value + SD. Statistical significance was determined by using t-test. CDC, Centers for Disease Control and Prevention; HA, hemagglutinin; JRC, Joint Research Centre; MP, matrix protein; NS, not significant;

In the first scenario, we added only HPAI H5N1 clade 2.3.4.4b RNA to the wastewater samples. The JRC-HA, JRC-MP, and CDC influenza A assays produced comparable viral RNA copy numbers, indicating detection near the analytical limits of the JRC assays (Figure 3, panel A). For the second scenario, we combined low concentrations (10 or 20 copies/µL) of HPAI H5N1 clade 2.3.4.4b RNA and seasonal influenza A RNA (35 copies/µL) to simulate periods of low seasonal influenza activity or low avian influenza prevalence. The JRC-HA and JRC-MP assays selectively detected HPAI H5N1 clade 2.3.4.4b RNA, whereas the CDC influenza A assay detected both avian and seasonal human influenza A RNA without distinction (Figure 3, panel B). 

In the third scenario, we added low concentrations (10 or 20 copies/µL) of HPAI H5N1 clade 2.3.4.4b RNA to a high concentration (3,500 copies/µL) of seasonal influenza A background, representing a possible early stage of HPAI detection during peak seasonal influenza activity. The JRC-HA assay detected HPAI H5N1 RNA without cross-reactivity, whereas the JRC-MP detected both avian and seasonal human influenza A RNA, but yielded lower copy number estimates than the CDC influenza A assay (Figure 3, panel C). Together, our results suggest that JRC-HA identifies and discriminates HPAI H5N1 clade 2.3.4.4b, whereas JRC-MP provided complementary detection but might lack specificity during high seasonal influenza A circulation.

## Conclusions

We propose the complementary use of CDC Flu SC2 and JRC-HA and JRC-MP duplex assays in a dual-duplex approach (Figure 4). The influenza A and B components of the CDC Flu SC2 multiplex assay are useful for routine communitywide surveillance of influenza A and B, ensuring continuity of the current public health reporting system. The JRC-HA and JRC-MP duplex assay, after the CDC Flu SC2 duplex, is used to specifically identify the occurrence of HPAI H5N1 clade 2.3.4.4b virus in a population, providing an early-warning capability that is effective in the presence of seasonal influenza backgrounds.

**Figure 4:**
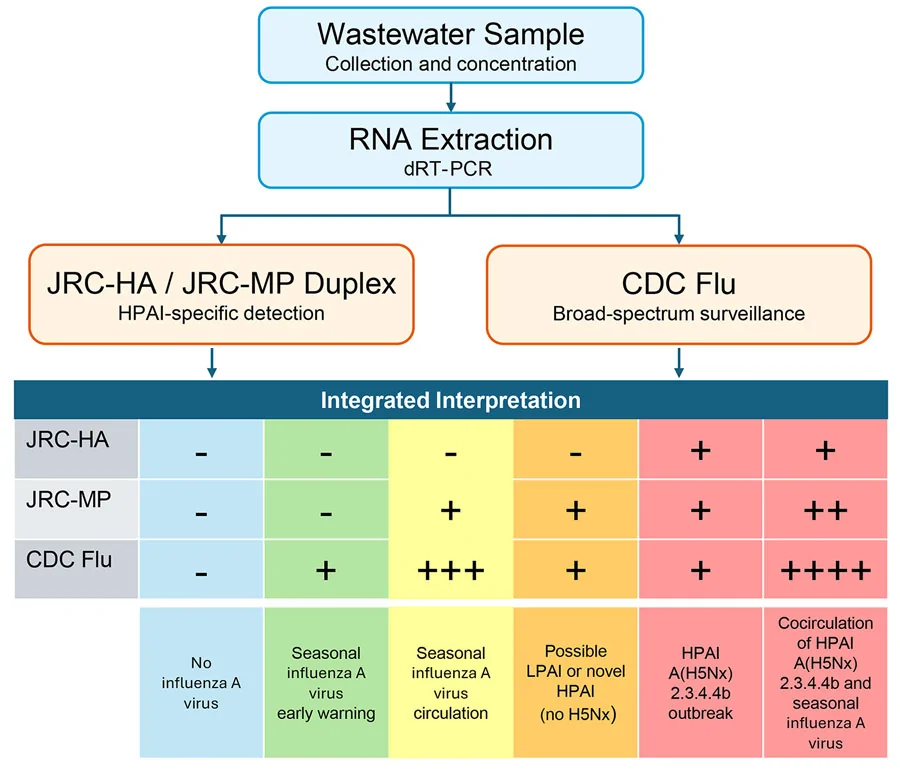
Interpretation of dual duplex dRT-PCR results for differentiating avian and seasonal influenza A viruses in wastewater. Extracted RNA from wastewater samples is analyzed by using the JRC-HA and JRC-MP duplex assay for detection of HPAI A(H5Nx) clade 2.3.4.4b viruses and the influenza A and B components of the CDC influenza and SARS-CoV-2 multiplex assay for broad influenza A virus surveillance. Results from both assays are then integrated to classify wastewater detections according to the presence, absence, and relative signal intensity of assay targets. Detection patterns are interpreted as indicating no influenza A virus detected, seasonal influenza A early warning, seasonal influenza A circulation, possible LPAI or non–clade 2.3.4.4b HPAI virus, HPAI A(H5Nx) clade 2.3.4.4b circulation, or co-circulation of HPAI A(H5Nx) clade 2.3.4.4b and seasonal influenza A viruses. Minus signs (−) indicate no target detection; increasing numbers of plus signs (+ to ++++) indicate increasing relative assay signal. CDC, Centers for Disease Control and Prevention; HA, hemagglutinin; HPAI, highly pathogenic avian influenza; JRC, Joint Research Centre hemagglutinin; LPAI, low pathogenicity avian influenza; MP, matrix protein; RT-dPCR, reverse transcription digital PCR.

We evaluated our dual duplex dRT-PCR by using animal-derived viral material and spiked wastewater RNA extracts, rather than confirmed HPAI-positive wastewater samples. Further field evaluation will be useful to assess performance under naturally occurring conditions and in different wastewater matrices. Because influenza viruses evolve rapidly, periodic sequence-based evaluations, followed by experimental validation and assay updates when necessary, will be required to maintain assay performance as new H5 clades emerge. 

A clade-specific wastewater result would not by itself determine a different public health response from a broad H5-positive result. However, it could reduce ambiguity, prioritize confirmatory testing, and enable integration of wastewater findings with veterinary, wildlife, domestic animal, and human surveillance data.

Appendix Additional information about differentiating avian and seasonal influenza viruses in wastewater by digital PCR
