# Supplementary material for: Differentiating Avian and Seasonal Influenza Viruses in Wastewater by Digital PCR
Source: Emerg Infect Dis. 2026 Nov;32(11):1879–84. doi: 10.3201/eid3211.261234 (PMC13715870; doi:10.3201/eid3211.261234)
Supplement: Supplementary file 1 [file 26-1234-Techapp-s1.pdf]

Article DOI: https://doi.org/10.3201/eid3211.261234

EID cannot ensure accessibility for supplementary materials supplied by authors. Readers who have difficulty accessing supplementary content should contact the authors for assistance.

Differentiating Avian and Seasonal Influenza Viruses in Wastewater by Digital PCR

Appendix

JRC-HA and JRC-MP assays (main features)

The JRC-HA and JRC-MP assays were previously developed and evaluated by Buttinger et al. (2025). JRC-HA targets a conserved region of the haemagglutinin gene associated with HPAI A(H5Nx) clade 2.3.4.4b, whereas JRC-MP targets a conserved region of the matrix gene and provides broader influenza A detection.

Both assays were optimized for real-time PCR and digital PCR and can be performed independently as simplex reactions or together in duplex format.

Primers and probes

Primers and probes of the JRC-MP/JRC-HA duplex assay and the Influenza A and B components of the CDC Flu SC2 multiplex assay used in this study are listed in Appendix Table 1.

PCR conditions

For real time qPCR analyses, the Quantstudio7 system (ThermoFisher) was used with the TaqPath 1-Step RT-qPCR Master Mix, in a final volume reaction of 25µl, comprising primers and probe according to the concentration reported in Appendix Table 1 and 4 µl of template. Nuclease free water was added to complete the volume.

The thermal profile is illustrated in Appendix Table 2. Thermal cycling was conducted according to the developer's protocol for both the CDC Flu SC2 multiplex assay (*1*) and for the JRC-HA/JRC-MP duplex assay (*2*) at the annealing temperature of 55°C and 60°C, respectively.

PCR curves were evaluated using QuantStudio Real-Time PCR software v1.7.2 and CT and end relative fluorescent units were exported as .csv files and processed in Excel.

For dPCR analyses, the QIAcuity Four 5-plex digital PCR system (Qiagen) was used with a 26K 24 wells nanoplate. Reaction employed the QIAcuity One-Step Advanced PCR kit (QIAGEN) in a final volume of 40 µL, comprising 10 µL of 4x One-Step advanced Probe Master Mix, 0.4 µL of 100x One-step Advanced RT Mix, primers and probe according to the concentration reported in Appendix Table 1, and 4 µL of template. Nuclease free water was added to complete the volume.

Thermal cycling conditions are illustrated in Appendix Table 3. For the CDC Flu SC2 multiplex assay, thermal cycling was conducted according to the developer's protocol, with an annealing temperature of 55°C. For the JRC-HA and JRC-MP duplex assay, annealing temperature was lowered at 56.4°C in comparison to previously published 60°C (*2*), to improve the separation between positive and negative partitions in the samples presenting two mutations in the region recognized by the probe. Representative plots are shown in Appendix Figure 2. Partitions were imaged with a 500 ms exposure time and a gain setting of 6 for both the green and yellow channels. The QIAcuity Software Suite version 3.1.0.0 was used to determine sample thresholds according to the manual global threshold approach, with thresholds established based on the fluorescence signals observed in the no-template controls (*3*).

JRC-HA and JRC-MP assays (summary of previous published results, Buttinger et al. 2025)

In-silico assessment

In-silico analyses were performed against 35,838 avian influenza A sequences, including 9,253 HPAI and 73 LPAI A(H5Nx) sequences collected worldwide. JRC-HA showed high predicted detection of HPAI A(H5Nx) clade 2.3.4.4b sequences, with limited or no predicted detection of other H5 clades under stringent conditions. In contrast, JRC-MP showed broader predicted reactivity across influenza A subtypes, consistent with the high conservation of the matrix gene.

At clade level, JRC-HA detected almost all available clade 2.3.4.4b sequences, whereas clade 2.3.2.1c and clade 2.3.4.4h sequences were not detected under standard

stringent conditions and clade 2.3.2.1a was only partially detected. JRC-MP showed broader detection of both HPAI and LPAI A(H5Nx) sequences.

Wet-lab assessment

In a blinded inter-laboratory analysis of 40 samples, JRC-HA detected all 26 HPAI A(H5N1) clade 2.3.4.4b isolates and none of the three LPAI H5 isolates, eight non-H5 influenza A isolates, or three negative controls. JRC-MP detected all 26 target isolates but also detected the other influenza A viruses included in the panel, confirming its broader influenza A reactivity.

Comparison with CDC-Flu A assay

The CDC Flu A assay detected both seasonal human influenza A and HPAI A(H5N1) clade 2.3.4.4b RNA. JRC-HA showed no detectable signal with the seasonal human influenza A or B controls and preferentially detected the avian H5 target. JRC-MP also detected seasonal human influenza A RNA, but with lower efficiency than CDC Flu A, while retaining stronger detection of HPAI A(H5N1) clade 2.3.4.4b RNA.

Wastewater performance

In Italian wastewater samples, JRC-HA and JRC-MP maintained specificity in the presence of wastewater RNA and showed good tolerance to matrix-associated inhibition. JRC-HA did not cross-react with wastewater samples containing seasonal human influenza A or B, whereas JRC-MP showed broader influenza A detection but lower sensitivity for seasonal human influenza A than the CDC assay.

Further evaluation of the JRC-HA and JRC-MP assays (this study)

In silico comparative analysis of H5 wastewater assays

Primer and probe sequences were retrieved for Wolfe-HA (*4*), CDC-HA (*5*), and JRC-HA/JRC-MP (*2*) assays, and an *in silico* analysis was performed to assess predicted cross-reactivity and clade specificity. For LPAI sequences, complete H5-annotated HA and MP sequences available in GISAID from January 2020 onward were screened. For the LPAI analysis, we applied a stringent criterion of a maximum of 2 mismatches per oligonucleotide to provide a conservative estimate of potential cross-reactivity For HPAI sequences, complete HA and MP sequences available in GISAID from August 2024 onward were analyzed. For the HPAI analysis, we used a more permissive threshold of a maximum of 5 mismatches per oligonucleotide (excluding only the last 2 bases of each oligonucleotide) to compare

predicted detection among closely related H5 clades and to assess discrimination between clade 2.3.4.4b.

Analysis of recent isolates from wild birds and poultry

The JRC-HA/JRC-MP duplex assay was evaluated using HPAI A(H5N1) clade 2.3.4.4b viruses collected from wild birds and poultry in northern Italy between October and December 2025. Samples used are described in Appendix Table 4. Sequence alignment between the primers and probes sequences of the JRC-HA and JRC-MP assays and the corresponding targeted regions in these viral isolates is shown in Appendix Figure 1.

JRC-HA and JRC-MP assay specificity and sensitivity at the annealing temperature of 56.4°C

To assess whether the specificity and sensitivity of the JRC-HA/JRC-MP duplex assay in RT-dPCR were maintained at temperature of 56.4°C, synthetic RNAs representing human influenza A, human influenza B, parainfluenza virus 1, parainfluenza virus 4, LPAI A(H3N2) (all from Twist Bioscience) and a HPAI A(H5N1) 2.3.4.4b positive RNA isolated from an infected animal (*Vulpes vulpes*) were tested using the JRC-HA/JRC-MP duplex assays. RT-dPCR conditions for the duplex assays were maintained as described above. The performance of the JRC-HA and JRC-MP duplex assay was compared with the CDC human influenza duplex assay (Flu A and Flu B).

To determine whether the JRC-HA/JRC-MP duplex assay maintained the sensitivity previously established at an annealing temperature of 60°C, with a reported limit of detection (LOD) of around 5 copies/reaction (*2*), low concentrations of HPAI A(H5N1) clade 2.3.4.4b RNA were tested. Specifically, 5 or 7 RNA copies per reaction were used as templates in ten independent reactions for each concentration, following the procedure described previously (*2*) and according to the ISO 20395:2019 (*6*). LOD is considered the concentration at which all the tested replicates are positive (presenting at least one positive partition). No positive partitions were detected in the negative (no-template) control.

JRC-HA and JRC-MP assay secondary-structure prediction

Using Primer3Plus thermodynamic secondary-structure prediction, the highest Tm among self-dimer, self-end, and hairpin structures across the JRC-HA and JRC-MP primers and probes was 44°C, well below the assays' 56.4°C annealing temperature. For this reason, stable primer-dimer or secondary structure formation under the reported reaction conditions was considered unlikely.

References:

1. Shu B, Kirby MK, Davis WG, Warnes C, Liddell J, Liu J, et al. Multiplex real-time reverse transcription PCR for influenza A virus, influenza B virus, and severe acute respiratory syndrome coronavirus 2. Emerg Infect Dis. 2021;27:1821–30. PubMed https://doi.org/10.3201/eid2707.210462

2. Buttinger G, Petrillo M, Valastro V, Marciano S, Crimaudo M, D'Amico V, et al. Novel (d)PCR assays for influenza A(H5Nx) viruses clade 2.3.4.4b surveillance. Euro Surveill. 2025;30:2500183. PubMed https://doi.org/10.2807/1560-7917.ES.2025.30.33.2500183

3. Dreo T, Pirc M, Ramšak Ž, Pavšič J, Milavec M, Zel J, et al. Optimizing droplet digital PCR analysis approaches for detection and quantification of bacteria: a case study of fire blight and potato brown rot. Anal Bioanal Chem. 2014;406:6513–28. PubMed https://doi.org/10.1007/s00216-014-8084-1

4. Wolfe MK, Duong D, Shelden B, Chan EMG, Chan-Herur V, Hilton S, et al. Detection of hemagglutinin H5 influenza A virus sequence in municipal wastewater solids at wastewater treatment plants with increases in influenza A in spring, 2024. Environ Sci Technol Lett. 2024;11:526–32. https://doi.org/10.1021/acs.estlett.4c00331

5. Centers for Disease Control and Prevention. National wastewater surveillance system analytics data set, year(s), as compiled from data provided through national wastewater surveillance system in the rapid response research and surveillance branch (R3SB). [cited 2026 Aug 14]. https://data.cdc.gov/Public-Health-Surveillance/CDC-Wastewater-Data-for-Avian-Influenza-A-H5-/mtpu-urpp

6. ISO 20395:2019, Biotechnology — Requirements for evaluating the performance of quantification methods for nucleic acid target sequences — qPCR and dPCR. International Organization for Standardization.

Appendix Table 1: primers and probes sequences and concentration used in PCR reactions

Assay	Oligo code	Oligonucleotide sequence (5′ to 3′)	Concentration in PCR reaction
JRC-HA (*2*)	HA01-For	ACTGGGCTCAGAAATAGTCCTC	900nM
	HA01-Rev	TCCCCTGCTCATTGCTATGATG	900nM
	HA01-Probe	FAM-AGAGGGAGGATGGCAGGGAATGGTT-QSY7	200nM
JRC-MP (*2*)	MP01-For	TGTCTTTGCAGGGAAGAACACC	900nM
	MP01-Rev	ACGGTGAGCGTGAACACAAATC	900nM
	MP01-Probe	VIC-ATCTTGAGGCTCTCATGGAATGGCT-QSY7	100nM
Influenza A (Flu A) (*1*)	InfA-For1	CAAGACCAATCYTGTCACCTCTGAC	400nM
	InfA-For2	CAAGACCAATYCTGTCACCTYTGAC	400nM
	InfA-Rev1	GCATTYTGGACAAAVCGTCTACG	600nM
	InfA-Rev2	GCATTTTGGATAAAGCGTCTACG	200nM
	InfA-Probe	6-FAM-TGCAGTCCTCGCTCACTGGGCACG-BHQnova	200nM
Influenza B (Flu B) (*1*)	InfB-For	TCCTCAAYTCACTCTTCGAGCG	800nM
	InfB-Rev	CGGTGCTCTTGACCAAATTGG	800nM
	InfB-Probe	HEX-CCAATTCGAGCAGCTGAAACTGCGGTG-BHQnova	200nM

Appendix Table 2: Thermal cycling conditions for RT-qPCR reaction

Step	Cycles	Temperature	Time
UNG Incubation	1	25°C	2 min
RT incubation	1	50°C	15 min
Enzyme activation	1	95°C	2 min
Amplification	45	95°C	15 s
		annealing °C	60 s

Appendix Table 3: Thermal cycling conditions for RT-dPCR reaction

Step	Cycles	Temperature	Time
RT incubation	1	50°C	40 min
Enzyme activation	1	95°C	2 min
Amplification	45	95°C	5 s
		annealing °C	30 s

Appendix Table 4: Bird samples collected from October to December 2025 during passive and active surveillance activities in wild and domestic birds in North-East Italy. The presence of HPAI (H5N1) 2.3.4.4b virus was confirmed by RT-qPCR and whole genomic sequencing methods in use at the EU and National reference laboratory for Avian Influenza and Newcastle Disease

				Genotype	
Sample ID	Sample	Matrix	GISAID Isolate ID	Genin	ggFLU
A	A/turkey/Italy/25VIR8933–13/2025	Tracheal Swab	EPI_ISL_20207397	EA-2024-DI DI.2.1	B.18
B	A/mallard/Italy/25VIR10761–1/2025	Cloacal Swab	EPI_ISL_20282404	EA-2024-DI DI.2.1	–
C	A/eurasian-wigeon/Italy/25VIR9558–3/2025	Tracheal Swab	EPI_ISL_20237305	EA-2024-DI DI.2.1	B.18
D	A/yellow-legged-gull/Italy/25VIR10550–1/2025	Organ pool	EPI_ISL_20282392	EA-2024-DI DI.2.1	–
E	A/mute-swan/Italy/25VIR10388–2/2025	Organ	EPI_ISL_20261558	EA-2024-DI DI.2.1	B.18
F	A/mute-swan/Italy/25VIR10598–1/2025	SNC	EPI_ISL_20282395	EA-2024-DI DI.2.1	–
G	A/chicken/Italy/25VIR10798–1/2025	Organ pool	EPI_ISL_20282406	EA-2024-DI DI.2.1	–
H	A/turkey/Italy/25VIR10392–1/2025	Tracheal Swab	EPI_ISL_20286945	EA-2024-DI DI.2.1	–
I	A/muscovy-duck/Italy/25VIR10762–1/2025	Cloacal Swab	EPI_ISL_20261564	EA-2024-DI DI.2.1	B.18
J	A/chicken/Italy/25VIR10912–4/2025	Tracheal Swab	EPI_ISL_20282387	EA-2024-DI DI.2.1	–

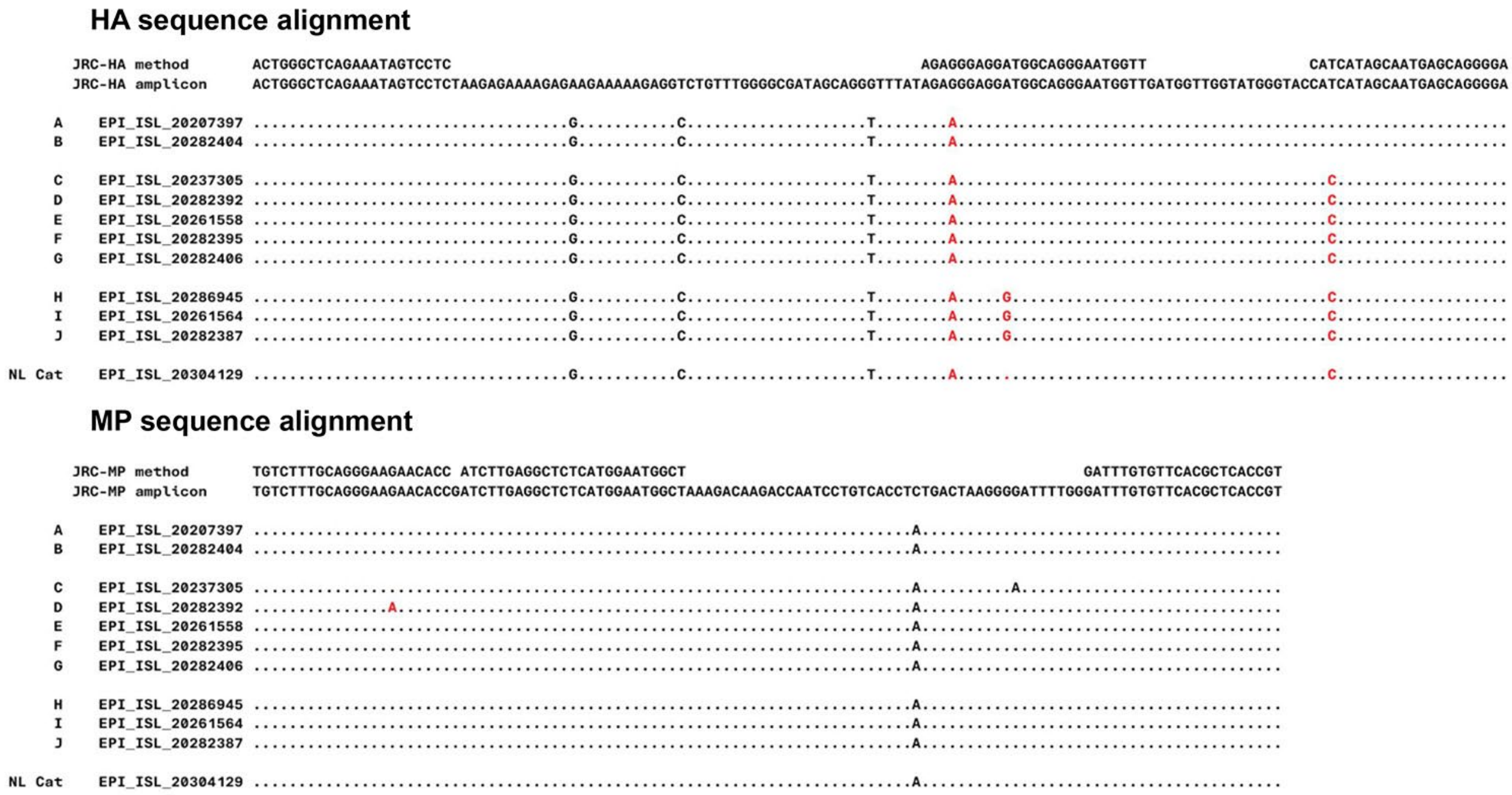

Appendix Figure 1. Sequence alignment of the JRC-HA and JRC-MP primer- and probe-binding regions with the reference amplicons, HPAI A(H5N1) clade 2.3.4.4b viruses A–J (Appendix Table 4), and an HPAI A(H5N1) clade 2.3.4.4b virus from a cat in the Netherlands. Oligonucleotide-binding site substitutions are shown in red; substitutions within the amplicons are shown in bold.

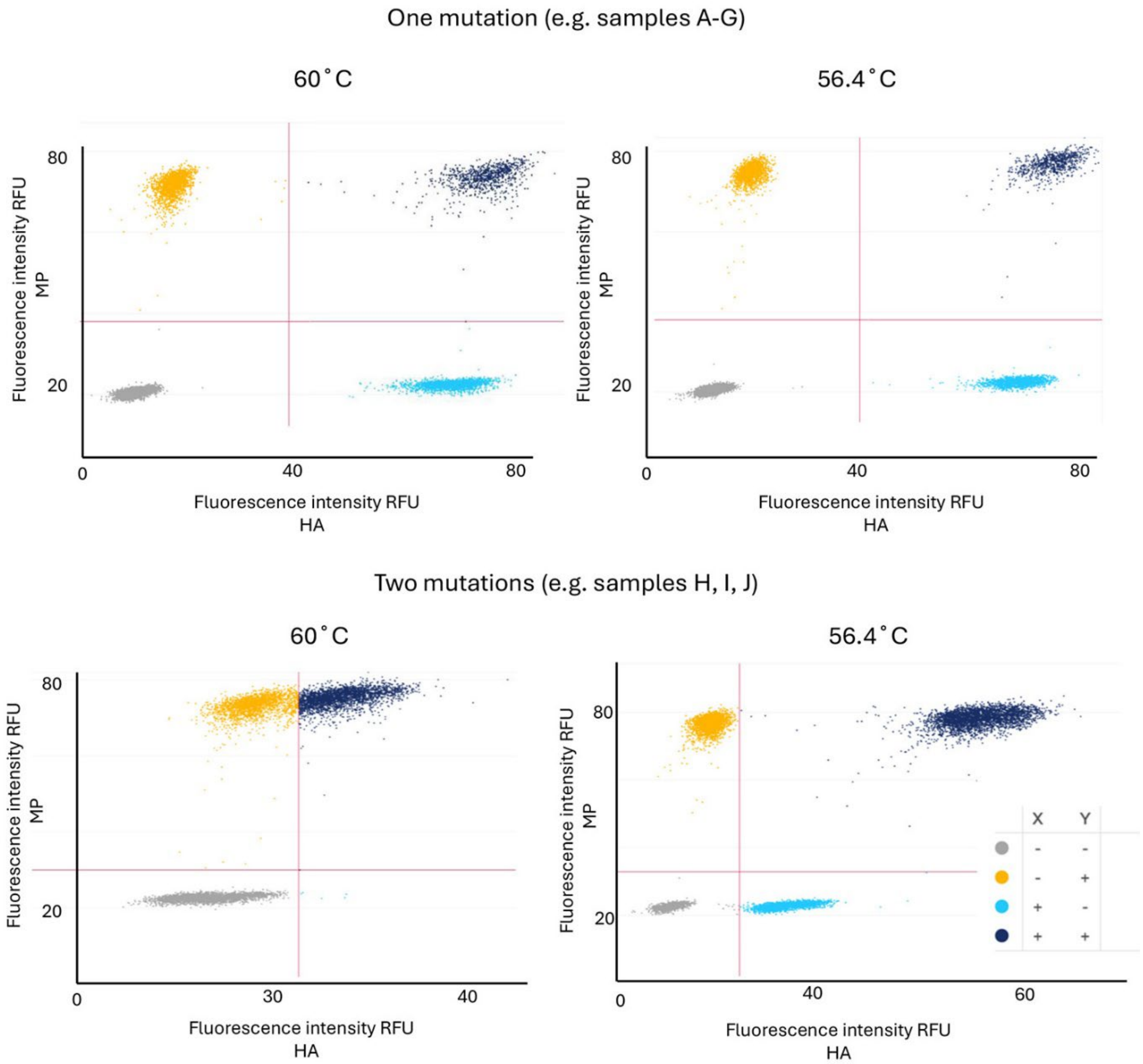

Appendix Figure 2. 2D scatterplot of duplex HA/MP dPCR. Representative two-dimensional fluorescence intensity plot showing the distribution of events according to HA (x-axis) and MP (y-axis) fluorescence. Horizontal and vertical thresholds (pink lines) divide the plot into four quadrants, corresponding to HA/MP double-negative (gray), HA-negative/MP-positive (yellow), HA-positive/MP-negative (cyan), and HA/MP double-positive (dark blue) populations. Number of mutations in the region recognized by the probe and annealing temperature used for dPCR are indicated at the top of the plots.
